# Talbot-Lau x-ray phase-contrast setup for fast scanning of large samples

**DOI:** 10.1038/s41598-018-38030-3

**Published:** 2019-03-12

**Authors:** Maria Seifert, Veronika Ludwig, Sebastian Kaeppler, Florian Horn, Pascal Meyer, Georg Pelzer, Jens Rieger, Daniel Sand, Thilo Michel, Jürgen Mohr, Christian Riess, Gisela Anton

**Affiliations:** 1 Friedrich-Alexander-University Erlangen-Nürnberg (FAU), Erlangen Centre for Astroparticle Physics, 91058 Erlangen, Germany; 2 Friedrich-Alexander-University Erlangen-Nürnberg (FAU), Pattern Recognition Lab, 91058 Erlangen, Germany; 3 Karlsruhe Institute of Technology (KIT), Institute of Microstructure Technology, 76344 Eggenstein-Leopoldshafen, Germany

## Abstract

Compared to conventional attenuation x-ray radiographic imaging, the x-ray Talbot-Lau technique provides further information about the scattering and the refractive properties of the object in the beam path. Hence, this additional information should improve the diagnostic process concerning medical applications and non-destructive testing. Nevertheless, until now, due to grating fabrication process, Talbot-Lau imaging suffers from small grating sizes (70 mm diameter). This leads to long acquisition times for imaging large objects. Stitching the gratings is one solution. Another one consists of scanning Talbot-Lau setups. In this publication, we present a compact and very fast scanning setup which enables imaging of large samples. With this setup a maximal scanning velocity of 71.7 mm/s is possible. A resolution of 4.1 lines/mm can be achieved. No complex alignment procedures are necessary while the field of view comprises 17.5 × 150 cm^2^. An improved reconstruction algorithm concerning the scanning approach, which increases robustness with respect to mechanical instabilities, has been developed and is presented. The resolution of the setup in dependence of the scanning velocity is evaluated. The setup imaging qualities are demonstrated using a human knee *ex-vivo* as an example for a high absorbing human sample.

## Introduction

Conventional x-ray attenuation radiography and tomography is the gold standard for several detection tasks in medical imaging and non destructive testing. Here, the image contrast is generated by the attenuation properties of the object. Nevertheless, in those standard x-ray systems, no information about the refractive properties (phase image) and the small angle scattering (dark-field image) due to microstructures of the object is gained.

In 1965 Bonse and Hart^1^ were the first who succeeded in developing an x-ray phase-contrast interferometer. Based on this work, in recent years many different methods have been established to get access to further information about a sample in addition to the attenuation properties. Besides the phase information, the dark-field image has been introduced as a third image type by Pfeiffer *et al*.^2^. Further investigations of the dark-field signal have been published for example by Rigon *et al*.^3^, Yashiro *et al*.^4^, Lynch *et al*.^5^, Strobl^6^ and Endrizzi *et al*.^7^.

These techniques can be divided into five main categories^8^: the interferometric techniques using crystals^9^, the propagation based methods^10,11^, the analyzer-based methods^12–14^, the grating interferometric methods^15–17^ and the grating non-interferometric methods^18–21^. Additionally, approaches using multiple phase-gratings have been made^22,23^. Especially the grating interferometric methods (Talbot-Lau imaging) and the grating non-interferometric methods (edge illumination) have shown promising results in medical imaging^24–30^ and non-destructive testing^31–37^. Furthermore, these two techniques could be operated using conventional x-ray sources^17,19^, which gives the opportunity to be adopted in clinics and industrial test facilities.

For the latter two techniques mentioned, micro-sized optical elements (which are called masks in edge illumination and gratings in Talbot-Lau interferometry) are needed which are placed within the beam path. Structures with high aspect ratio are required. The deep x-ray LIGA process^38^ is performed to access the high aspect ratio needed; but the structured area is limited to 70 mm diameter or 50 × 50 mm^2^ in a square. Larger areas (20 × 20 cm^2^) can only be achieved with a complex stitching process of 16 single gratings^39–41^. Hence, due to magnification of the setup only small fields of view can be obtained in the object plane. In case of large objects usually small regions of interest of some square centimeters are separately acquired and are combined afterwards to a large image. Furthermore, the phase-stepping procedure^16^ which is necessary in grating-based interferometric methods to resolve the sub-pixeled information is time-consuming and has high mechanical requirements to move the gratings very precisely^30,42–45^. To bring x-ray phase-contrast imaging to clinics and non-destructive testing workflows, a highly stable system with a large field of view and a fast acquisition time has to be available.

To overcome these problems in recent years several scanning setups have been proposed^46–48^. In these setups the limitation due to the grating area is reduced to one dimension as in the second dimension a scanning procedure is performed. To achieve large fields of view perpendicular to the scanning direction several gratings have been mounted and aligned in this direction, perpendicular to the scanning direction. Such gratings are called line gratings in the following. In this context, different approaches have been published, for example an edge illumination scanning system^49^ and Talbot-Lau imaging scanning systems^50,51^ which show promising results. In a scanning setup object and gratings are moved relatively to each other (see Fig. 5). Hence, the field of measurement in scanning direction is no longer restricted by the size of the gratings. Gromann *et al*.^50^ imaged successfully a porcine thorax in one scanning procedure.

In conventional Talbot-Lau setups the interference pattern is sampled by moving a grating in front of the detector. This procedure is called phase-stepping^16^. In Talbot-Lau scanners the phase-stepping of the grating is replaced by the object movement relatively to the fringe pattern which is created by a slight misalignment of the gratings^46–48,52^.

The basic principle of a Talbot-Lau interferometer is described in many publications, such as by Weitkamp *et al*.^16^, Pfeiffer *et al*.^17^, and will not be detailed here. Roessl *et al*.^53^ summarized general considerations regarding the comparison of scanning Talbot-Lau systems and full-field 2D setups.

In this publication, we present our new Talbot-Lau grating scanner setup, developed for large samples, using a high scanning speed and requiring only a low dose for good quality imaging. With our setup objects of a size of 17.5 × 150 cm^2^ can be imaged with one scanning procedure. Whereas the 17.5 cm are limited by the detector size and the 150 cm by mechanical reasons of the linear stages which move the object. To be able to image high absorbing human tissue or some industrial samples, the design energy chosen is 60 keV. A further aim was the minimization of the scanning time. Bachche *et al*.^51^ scanned objects with a scanning velocity of 5 mm/s, Astolfo *et al*.^49^ used a scanning velocity of 2.5 mm/s and Gromann *et al*.^50^ of around 9 mm/s. The scanning velocity of the presented setup is only limited by the read out of the detector. A maximal frame rate of 840 frames per second can be achieved with the line detectors. This corresponds to a maximal scanning velocity of 71.7 mm/s in the object plane. Of course, it has to mentioned that image noise increases by decreasing the scanning time as the power of the x-ray source is limited.

The scanning and reconstruction algorithm is based on the algorithms presented by Kottler *et al*.^46^, and Koehler *et al*.^48^. However, slit-scanning setups are, due to the large grating sizes, particularly susceptible to moiré artifacts which arise from phase changes due to grating vibrations. To improve robustness with respect to these artifacts we extend the reconstruction algorithm by including compensation terms. Hence, the presented reconstruction algorithm for slit-scanning systems is capable of suppressing moiré artifacts.

Firstly, the resolution of the system in dependence of different scanning velocities up to 71.7 mm/s is evaluated by imaging a Siemens star and a sharp edge of an alumimium sheet. This gives information about the spatial resolution of the system.

Secondly, to demonstrate the feasibility of the presented system for high-energy applications, a human knee *ex-vivo* has been measured. In this context, we examine the quality of imaging with a scanning velocity of 71.7 mm/s which corresponds to a dose of about 43 μGy air kerma.

## Results

### Resolution

The resolution of the scanning system is evaluated in dependence of the scanning velocity. Bachche *et al*.^51^ observed that the resolution in scanning direction can degrade clearly. In their setup a scanning velocity of 5 mm/s corresponds to a velocity of 1 pixel per frame. In our setup, we can adapt the detector’s number of frames per second to different scanning velocities. This dependency is also optimized in such a way that the object shift corresponds to one (binned) pixel per frame. As we perform a 2 × 2 binning, the object moves 198 μm between two frames in the detector plane. We chose three different scanning velocities to evaluate blurring and distortions. The chosen parameters are shown in Table 1. It has to be mentioned that the exposure time refers to the exposure time of one frame. One phase-stepping curve comprises 34 frames (page 7 Methods).

**Table 1:** Parameters of the measurements concerning the evaluation of blurring in dependence of the scanning velocity.

Exposure time [ms]	Frames per second	Scanning velocity [mm/s]
20.0	50	4.3
10.0	100	8.6
1.2	840	71.7

All measurements were performed at 60 kVp and 200 mA. The exposure time is inversely proportional to the scanning velocity; thus the photon statistics decreases with increasing scanning velocity while the exposure time is adapted to the scanning velocity.

**Figure 1:**
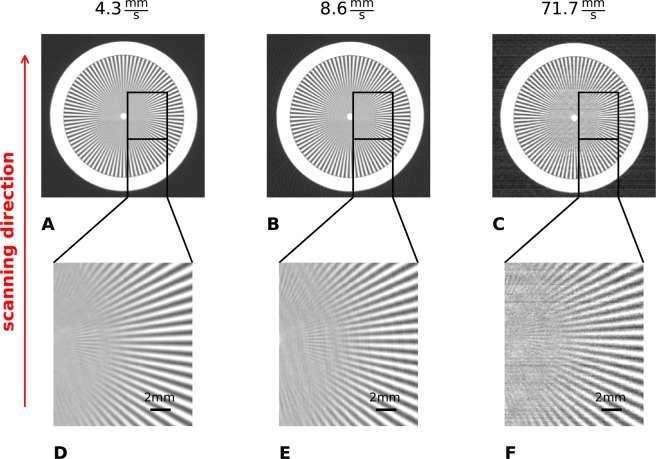
(**A**–**C**) Attenuation images of the Siemens star for different velocities. (**D**–**F**) Show zoomed parts of the Siemens star for better comparability.

In a first step, we image a Siemens star in order to evaluate the spatial resolution in dependence of the scanning velocity. The star has a diameter of 45 mm and is parted in steps of 2°. The stripes are made of 0.05 mm lead. The attenuation images for the different velocities are shown in Fig. 1(A–C). For better comparability a part of the star is shown in an enlarged version in Fig. 1(D–F).

The attenuation images demonstrate, that the star is not distorted due to the faster velocity. The circular shape can be reproduced for all measurements. As expected the noise increases with increasing velocity. Nevertheless, the resolution does not become worse for faster scanning velocities. This can be confirmed regarding the enlarged parts of the star. The highest frequency which can be resolved independently of the scanning velocity is around 4.1 lines/mm in the object plane.

**Figure 2:**
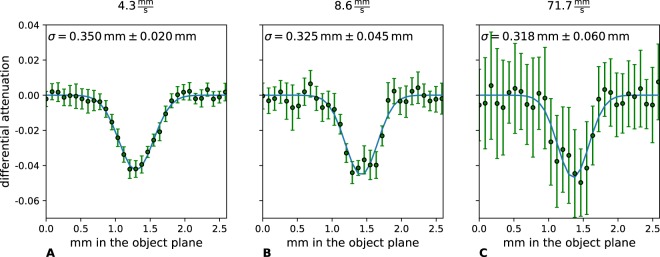
Lineplots of the attenuation image derivation of an aluminium sheet edge (green dots). The errorbars show the standard deviation of one measurement point over 20 lines to give an impression about the variance of the measurement over the lines. The blue line shows the Gaussian fit. The standard deviation *σ* of the Gaussians and its corresponding error concerning the 95% confidence interval is written on the top of the corresponding plot. The goodnesses of the fits (*χ*^2^) are (**A**) 0.091, (**B**) 0.389 and (**C**) 0.186. The errorbars show the standard deviation over 20 lines.

In a second step, we evaluate the resolution of an edge in the attenuation image, in analogy to the experiment of Bachche *et al*.^51^. For this purpose, we image the edge of an aluminium sheet and calculate the width by computing the derivative of the attenuation image intensity function. We perform a Gaussian fit to the derivations of the edges and determine the sigma values *σ*. The goodness of the fits are given as reduced *χ*^2^ values in the caption of Fig. 2. *χ*^2^ is defined as following:

$${\chi }^{2}=\frac{1}{k-p}{\sum }_{i=1}^{k}{(\frac{{y}_{i}-f({x}_{i})}{{s}_{i}})}^{2},$$

with *k* the number of all measurement points, *p* the number of fit-parameters (here 3 parameters for the Gaussian fit), *y*_*i*_ one measurement point, *f*(*x*_*i*_) the corresponding fitted value and *s*_*i*_ the standard deviation of each measurement point over 20 lines. The edge detection with first order derivative approximation and the Gaussian fits are depicted in Fig. 2. The green dots show the measurement values, the blue line the Gaussian fit, respectively. The errorbars show the standard deviation of one measurement point over 20 lines. Thus, an impression about the influence of the noise can be gained.

It can be observed that the standard deviation *σ* is about 4 pixels in the object plane for all three scanning velocities. Thus, obviously no blurring occurs in dependence of the sample’s velocity. Such successful reconstruction needs a good synchronization between detector read-out time and velocity. Of course, like in the measurement of the Siemens star the noise of the fast scanning image, which is shown in Fig. 2(C) is high compared to the other images. This is again due to the very short acquisition time which is necessary to fulfill the condition of shifting the object one pixel per frame.

### Imaging - Exemplary case study using a human knee *ex-vivo*

Horn *et al*.^30^ presented images of a human knee acquired with a high energy setup. Here, a tube voltage of 70 kVp was applied. It could be shown that especially in the dark-field image medical diagnosis of calcifications is possible^30^. Nevertheless, the acquisition process suffered from a small field of view of only about 20.9 × 22.6 mm^2^. Thus it took about 20 min to image the whole knee and the final image is composed of more than 90 single ones with a minimal dose of 0.14 mGy air kerma.

Here, we present the measurement of another human knee *ex-vivo* which is scanned with the high energy presented setup. The knee is imaged with two different scanning velocities. The parameters are shown in Table 2. All measurements have been carried out with a tube voltage of 70 kVp and using a 0.3 mm copper filter. The exposure time of one frame is adapted to the scanning velocity, like in the other measurements presented in this publication. The dose was measured with a DC300 ionization chamber of IBA dosimetry. It is comparable or lower than the dose of knee imaging used in conventional radiography systems as it is published by Huda *et al*.^54^ and Kwang *et al*.^55^ who state doses of 687 μGy for an adult patient and 278 μGy for slot-scanning radiography, respectively.

**Table 2:** Parameters of the measurements.

Exposure time [ms]	Tube current [mA]	Frames per second	Scanning velocity [mm/s]	Air kerma [μGy]	Scanning time [s]
20.0	127	50	4.3	250	27.8
1.2	385	840	71.7	43	1.6

**Figure 3:**
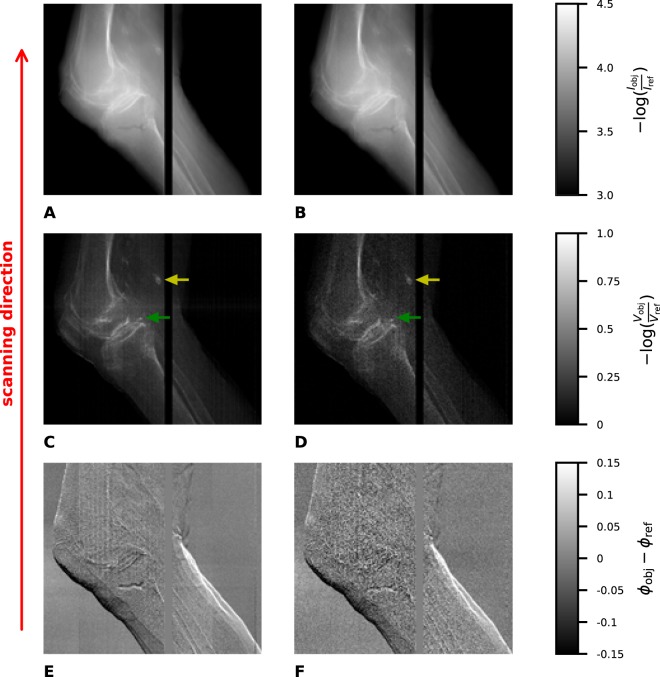
Attenuation images (**A**,**B**), dark-field images (**C**,**D**) and differential phase-contrast images (**E**,**F**) of a human knee, with 27.8 s (**A**,**C**,**E**) and 1.6 s (**B**,**D**,**F**) and 250 μGy (**A**,**C**,**E**) and 43 μGy (**B**,**D**,**F**), respectively. The black gap in the images is due to the gap between both line detectors. The yellow and the green arrow in the dark-field images point onto a not specified calcification which can be seen in both images. It has to be mentioned that the images only show parts in which the knee can be seen. The field of measurement is even larger.

The results of the measurements of the knee are depicted in Fig. 3. The black gap in the images is due to a gap between the single line detectors which are stitched together in the presented setup (further explanation see Methods). (A,B) show the attenuation images with different scanning velocities and, therefore, with different dose values. (C,D) show the dark-field images and (E,F) the differential phase-contrast images. It can be seen that the noise is much higher in the low dose images (B,D,F), especially in the dark-field image and the differential phase-contrast image. The joint space is clearly visible in the dark-field images. Furthermore, two small calcifications are visible in both dark-field images. They are marked by a yellow and a green arrow. The larger one which is marked by the yellow arrow can also be seen in both attenuation images. Nevertheless, the smaller calcification is hardly visible in one of the attenuation images.

**Figure 4:**
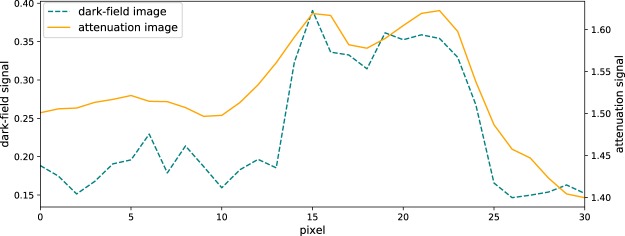
Lineplots through the calcification marked with the yellow arrow in Fig. 3 in the higher dose images. The blue line shows the lineplot of the dark-field image (Fig. 3(C)), the yellow one the lineplot of the attenuation image (Fig. 3(A)).

In Fig. 4 lineplots through the larger calcification in the attenuation and dark-field higher dose images which is marked with the yellow arrow in Fig. 3(C) are shown. The Michelson contrast which is defined as

$${C}_{{\rm{Michelson}}}=\frac{{S}_{{\rm{calcification}}}-{S}_{{\rm{tissue}}}}{{S}_{{\rm{calcification}}}+{S}_{{\rm{tissue}}}},$$

where *S*_calcification_ is the signal of the calcification in the corresponding image modality and *S*_tissue_ of the surrounding tissue, respectively, can be calculated. This contrast value is the same for the higher and the lower dose images: *C*_Michelson,dark−field_ = 0.3 for the dark-field image and *C*_Michelson,attenuation_ = 0.03 for the attenuation image.

## Discussion

We could show that a scanning velocity of up to 71.7 mm/s does not lead to a blurring of the acquired images. A resolution of $4.1\,\frac{{\rm{lines}}}{{\rm{mm}}}$ can be achieved. Thus, a maximal scanning velocity which is about ten times higher compared to existing scanning setups is possible. The spatial resolution keeps approximately constant varying the velocity. Nevertheless, noise of course increases with higher velocities especially in the dark-field and in the differential phase-contrast images as the exposure time per frame has to be reduced for such fast acquisitions. We could not increase the current of the tube anymore to enhance the photon statistic as we were at the maximum of the tube’s power limit. Thus, it would be advisable to use an x-ray tube which can provide more power. Additionally, it has to be taken into account that the G0 grating has a duty cycle of 0.6 which leads to more absorption of the grating than a grating with a conventionally used duty cycle of 0.5. Hence, the photon statistics is further reduced. Nevertheless, the choice of this duty cycle leads to higher visibilities over a wide range of energies^56^. This again reduces the noise in dark-field images. It has also to be taken into consideration that the absorbing effect of the G0 is in front of the patient, and de facto does not increase the delivered dose. Hence, it has to be found a compromise between a high visibility and a reduction of the x-ray photon statistic at the detector due to the optical elements. Further studies should show if improvements of interferometer specifications are possible and if the visibility can be further increased.

As an exemplary case study a human knee *ex-vivo* was successfully imaged with our scanning setup. The overall scanning time of the knee could be reduced to a minimum of about 1.6 s, which resulted in a dose comparable to the one received by a clinical attenuation radiography of 43 μGy air kerma. It has again to be mentioned that noise in the dark-field image and the differential phase-contrast image is increased for the fast scanning application. Nevertheless, a calcification which could be observed in the image which has been acquired in 27.8 s can also be seen in the low dose image. The Michelson contrast of the larger calcification is ten times higher in the dark-field images than in the attenuation images. Thus, we could show, that it is possible to get medically interesting images even for the fast scanning application. Momose *et al*.^28^, Nagashima *et al*.^57^ and Horn *et al*.^30^ imaged the joint space of a hand and a knee respectively and could show that additional information about potentially pathologic structures can be gained inter alia by the dark-field image. For example, as presented by Horn *et al*.^30^ chondrocalcinosis can be diagnosed in the dark-field image of the joint space. Also in the images presented in this publication the joint space is clearly visible in the dark-field images of both dose applications. This gives the opportunity for further examinations. It would be for example interesting to image knees with specific diseases, to check which diseases can be diagnosed in the images, acquired with our fast scanning setup.

To our knowledge the presented scanning setup is the fastest one published until now, combining a large field of view and an easy alignment process. The gratings can be aligned as one compact system; the precision of manually stitching being sufficient in this case. No complex stitching process is necessary. Thus, the whole system is easy to implement and does not need several motion stages for each single grating tile. It has to be mentioned, that the differential phase-contrast and the dark-field image are only sensitive for structures which cause refraction or scattering perpendicular to the grating bars. In the presented setup the grating bars are perpendicular to the scanning direction. Thus, only scattering and refraction in scanning direction can be detected. If the grating bars are rotated such that they are aligned in scanning direction in order to adapt the sensitive direction, the stitching process is much more complicated.

Furthermore, due to the cone beam shadowing has to be taken into account if the field of view is enlarged. These effects can be compensated by bending the gratings. Nevertheless, in the presented setup the influence of the shadowing effects is not as severe due to the orientation of the grating bars (see page 7 Methods) and no bending is necessary. If the gratings are aligned 90 degrees rotated, shadowing effects have to be considered.

The field of view is no longer restricted by the size of the gratings. It depends on the area the x-ray source is able to illuminate and on the size of the chosen line detector respectively the number of line detectors combined to one large detector. Combining several line detectors like in the presented setup, of course, the gap between the detectors (further explanations see section: page 7 Methods) should be avoided. If there are no detectors without margin available, the gap can be avoided by placing the detectors directly behind each other with a slight overlap between them. This leads to a more complicated aligning process as the gratings should show the same overlap as the detectors; they have to be placed parallel to the corresponding detector concerning the X-ray source. The advantage of line detectors over 2D flat panel detectors is the faster read-out time which enables such high scanning velocities. Of course more detector lines would increase the statistics of the phase-stepping curve but it would also slow down the whole scanning process due to the longer read-out time.

To conclude, with respect to medical applications we could show that it is possible to acquire fast scanning images which reveal more details than using the conventional attenuation radiography. The possibility of very fast scanning acquisitions gives the opportunity to reduce and to avoid motion artifacts, while the improved reconstruction algorithms suppresses moiré artifacts, which have previously been observed at slit-scanning setups. Hence, there are no restrictions anymore concerning the field of view due to the grating area as it is no problem to stitch several smaller gratings.

## Methods

### Setup

**Figure 5:**
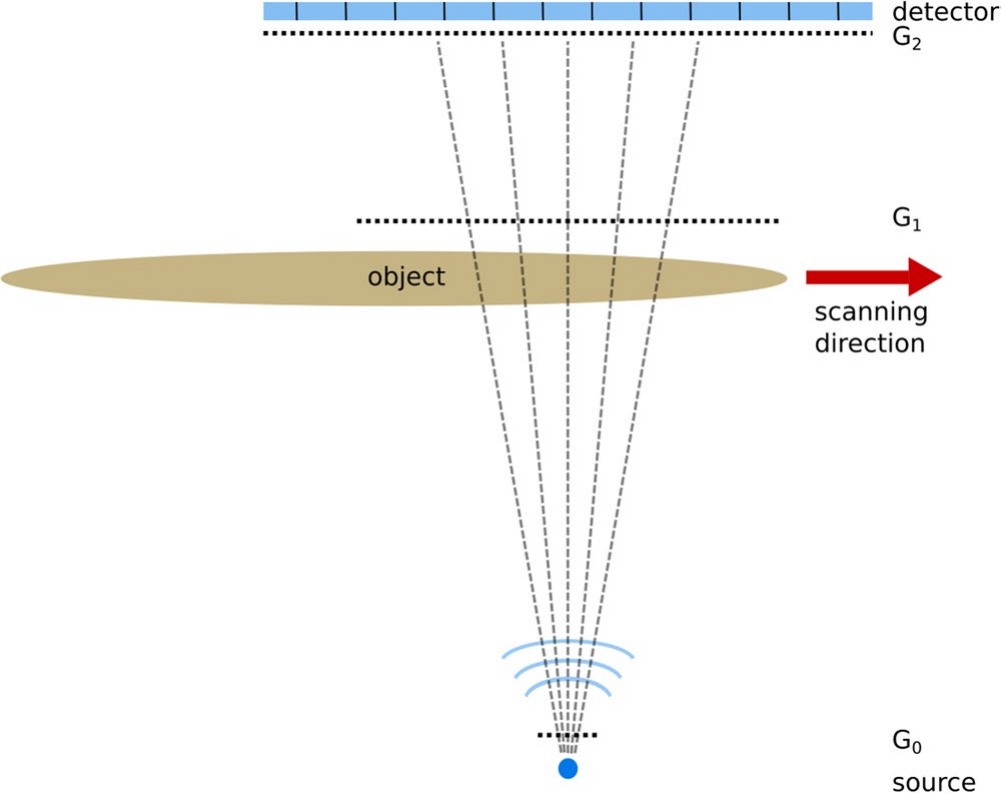
Schematic sketch of the scanning setup. The distances are not to scale.

The scanner system consists of a conventional Talbot-Lau setup which is made up of three gratings (Fig. 5)^17^.

**Figure 6:**
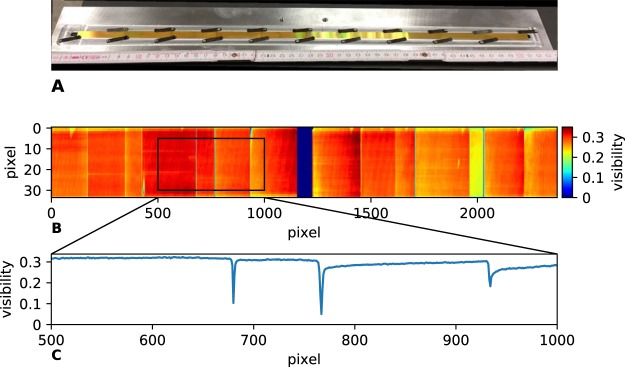
(**A**) Photography of the stitched G2 grating, (**B**) visibility map of a detector read out, and (**C**) zoom of the lineplot of the visibility of the mean over all detector lines.

The parameters of those gratings are listed in Table 3. The parameters are optimized according to Rieger *et al*.^56^. In contrast to a conventional grating setup two 5 × 5 cm^2^ gratings G1 and G2 are cut into stripes of 5 × 1 cm^2^ and are manually stitched on the short side. The gratings are fixed with clamps (Fig. 6(A)). The grating bars are orientated parallel to the long side of the stripes. Thus, no bending of the gratings is necessary as shadowing effects only occur in the direction, perpendicular to the grating bars. As in scanning direction the line detector comprises only 6.7 mm, shadowing effects can be neglected. After stitching the gratings they cover a size of 50 × 1 cm^2^ and each grating consists of 10 subgratings.

**Table 3:** Parameters of the gratings.

	G0	G1	G2
Material	Au	Au	Au
Period [m]	13.31	5.71	10.00
Height of bars [m]	200.0	6.3	200.0
Duty cycle	0.66	0.30	0.50
Distance from source [mm]	190	1130	1848

The distance of the source to the object comprises 800 mm. The magnification factor of the object plane into the detector plane is 2.3.

A conventional medical x-ray source is used: Siemens MEGALIX Cat Plus 125/40/90-125 GW. The whole detection system consists of two line detectors (Teledyne Dalsa Xineos-2301) which are placed side by side. Due to the inactive edges of both detectors there is an inactive zone of about 14.5 mm in between the detectors which results in a gap of 6.3 mm in the object plane. One detector comprises an area of 228 × 6.5 mm^2^ and has a pixel size of 99 μm. Thus, the field of view in the object plane including the gap between both detectors is about 17.5 cm. The object can be moved 150 cm transverse to the grating bars. The maximal number of frames per second (fps) is 840 fps in combination with 2 × 2 binned detector pixels.

The mean visibility at 60 kVp is 28.5% with a standard deviation of 2.9%. This proves that without any complicated aligning process of each grating tile, a homogeneous visibility distribution can be achieved. The visibility map and a mean over all detector lines can be seen in Fig. 6(B,C), respectively. The gap in visibility in the middle of the map is due to the interactive area between both detectors. The edges of neighboring subgratings also lead to drops in visibility. Those gaps comprise around 6 pixels which corresponds to a gap size of around 0.5 mm in the object plane. Nevertheless, even in those gaps the visibility never drops to zero. Thus, for each pixel a dark-field signal can be measured.

### Reconstruction procedure

#### Reconstruction method

Our reconstruction method is based on the approach presented by Koehler *et al*.^48^. However, this method does not account for grating motions during the scanning procedure^58^. Phase changes due to grating motions produce moiré artifacts in the reconstructed images. These artifacts greatly reduce image quality. We therefore modified the approach by Koehler *et al*.^48^ with a correction for grating motion. This is, to our knowledge, the first artifact correction algorithm proposed for slit-scanning systems.

In accordance with the images in our paper, we set the *x*-axis along the long side of the detector, i.e. along the detector lines or rows, whereas the *y*-axis is set along the scanning direction or the detector columns. Pixels with indices $(\tilde{x},\tilde{y})$ reside in the object coordinate system at the detector position, whereas pixels with indices (*x*, *y*, *n*) reside in the detector coordinate system and correspond to the *n*-th detector read out. *M*(*x*, *y*, *n*) describes the measured intensities during scanning. The number of detector pixels in scanning direction (after binning) is denoted as *N*_*y*_, with each pixel having a size of *s*_*y*_ in scanning direction. The scanning velocity is denoted by *v* and the detector readout rate is *r*. Before scanning, we acquire a reference scan over the whole detector using standard phase stepping^16^. We denote *I*_ref_(*x*, *y*), *V*_ref_(*x*, *y*) and *ϕ*_ref_(*x*, *y*) as the reference intensity, visibility and phase at detector coordinates (*x*, *y*). Thereby, the visibility is defined as *V* = *A/I* with *A* the amplitude of the phase-stepping curve and *I* the mean intensity. We use the same reference information for the whole scan. The reconstruction problem to solve for each point in the object coordinate system is given by

$$\begin{array}{c}\mathop{{\rm{argmin}}}\limits_{\begin{array}{c}T(\tilde{x},\tilde{y})\\ D(\tilde{x},\tilde{y})\\ \delta {\varphi }_{{\rm{o}}{\rm{b}}{\rm{j}}}(\tilde{x},\tilde{y})\end{array}}\,\sum _{k=0}^{{N}_{y}-1}[M(x,k,\frac{\tilde{y}\cdot {\rm{\Delta }}t}{v}+k)-{I}_{{\rm{ref}}}(\tilde{x},k)\cdot T(\tilde{x},\tilde{y})\cdot (1+{V}_{{\rm{ref}}}(\tilde{x},k)\\ \,\,\,\cdot D(\tilde{x},\tilde{y})\cdot \,\cos ({\varphi }_{{\rm{ref}}}(\tilde{x},k)+\delta {\varphi }_{{\rm{obj}}}(\tilde{x},\tilde{y})+\gamma (\tilde{x},k,\frac{\tilde{y}\cdot {\rm{\Delta }}t}{v}+k))){]}^{2},\end{array}$$

where $T(\tilde{x},\tilde{y})=\frac{{I}_{{\rm{obj}}}(\tilde{x},\tilde{y})}{{I}_{{\rm{ref}}}(\tilde{x},\tilde{y})}$, $D(\tilde{x},\tilde{y})=\frac{{V}_{{\rm{obj}}}(\tilde{x},\tilde{y})}{{V}_{{\rm{ref}}}(\tilde{x},\tilde{y})}$ and *δϕ*_obj_ denote the non-logarithmic object attenuation, the non-logarithmic dark-field and the differential phase at object coordinates $(\tilde{x},\tilde{y})$. In the images presented above the logarithmic attenuation and the logarithmic dark-field are shown. In order to avoid unnecessary interpolation we constrain the readout rate and velocity to *v* = *s*_*y*_·*r* such that the indices in above formula are always evaluated to integer quantities. It has to be emphasized that the velocity *v* in this case is the velocity calculated in the detector plane whereas the velocity given in the experimental part of the publication corresponds to the velocity in the object plane with respect to the magnification. The optimization problem can, after linearization, be solved using standard linear least squares methods. The difference between our objective function and that used in Koehler *et al*.^48^ is the addition of the phase correction term *γ*, which accounts for phase changes due to gratings motions. For *γ* = 0 both methods are identical.

#### Phase correction model

For phase stepping reconstruction, several approaches to estimate such a correction factor have been proposed^44,45,59–61^. These approaches have one shared assumption, namely that the grating motion occurs only perpendicular to the direction of the gratings bars. Under this assumption, the correction factor is identical for all detector pixels, i.e. *γ*(*x*, *y*, *n*) = *γ*(*n*).

However, more general motions, such as grating rotations, lead to phase changes which are position dependent. These phase changes are well approximated using a polynomial model that depends on the detector position^42,62^. For small grating setups, these rotations typically only become significant on larger time scales, such that the above mentioned assumption of a spatially invariant correction factor is usually sufficient to account for motion between individual phase-steps. However, the large stitched gratings of our scanning setup are particularly sensitive to rotation. Hence, we adopt the polynomial model for the long x-axis of the detector. For the short y-axis, we assume a constant phase correction in accordance to previous work for small gratings. These considerations yield the following model of the phase correction term *γ*(*x*, *y*, *n*) = *a*(*n*)·*x*^2^ + *b*(*n*)·*x* + *c*(*n*). Using this model, we hence need to estimate a set of three polynomial coefficient for each detector read out prior to performing image reconstruction.

#### Estimation of phase correction

Our estimation of the correction term is based on the fact that our setup is able to acquire an artifact-free reference scan through the use of continuous phase-stepping and a high detector read-out rate. We correlate the reference scan to each detector column at each read-out to estimate the change in phase, and then aggregate the results to compute the polynomial coefficients. To this end, we first generate a set of 40 equidistant ‘virtual’ phase-steps *P*(*x*, *y*, *s*) in the interval *s* ∈ [−*π*; *π*] using the reference scan:

1 $$P(x,y,s)={I}_{{\rm{ref}}}(x,y)\cdot (1+{V}_{{\rm{ref}}}(x,y)\cdot \,\cos ({\varphi }_{{\rm{ref}}}(x,y)+s))\,{\forall }_{s}.$$

Afterwards, we compute the correlation coefficient *C*(*y*, *n*, *s*) between each detector column and the corresponding phase-step column for each detector read out and each phase stepping position:

2 $$C(x,n,s)=\mathop{{\rm{correlation}}}\limits_{y}(M(x,\cdot ,n),P(x,\cdot ,s))\,{\forall }_{x,n,s}\,,$$

where · selects all elements along the y-axis. Assuming that the object signal is uncorrelated to the reference signal, we expect the highest correlation at the correct phase. Hence, we select the phase-step with the highest correlation as an intermediate correction term

3 $${\gamma }_{C}(x,y,n)=\mathop{{\rm{argmax}}}\limits_{s}\,C(x,n,s)\,{\forall }_{x,n}.$$

However, we have previously assumed that the phase drift is due to grating rotations. Hence, the correction term should correspond to our polynomial model. In a last step, we fit the above described 2nd-order polynomial model to the correction term of each detector read out to smooth the phase correction along the x-direction:

4 $$\gamma (x,y,n)={\rm{polyfit}}\,{\gamma }_{C}(x,n,s)\,{\forall }_{n}.$$

**Figure 7:**
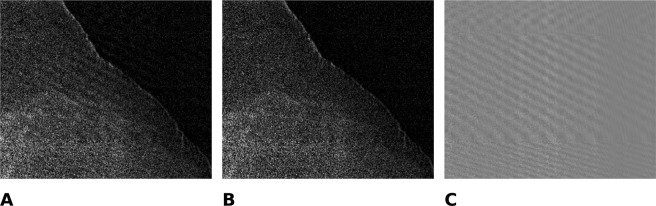
Effect of the proposed phase drift correction on the dark-field image of the knee specimen. (**A**) without correction, (**B**) with correction, (**C**) difference image showing the removed moiré artifact.

In order to increase robustness of the fit at object edges, which like artifacts also affect the phase, we use a robust bisquare loss function^63^ for the fit. After determining the correction term, the signal reconstruction is performed as described above. The effect of the proposed correction is illustrated in Fig. 7.

### Sample preparation and ethics statement

The measurement of the human knee *ex-vivo* has been performed as a feasibility study for imaging large, human samples with a fast scanning device. The use of the human sample for this experiment has been approved by the Ethics Committee of the University of Erlangen-Nuremberg (Germany). The tissues were used with the body donator’s written and informed consent. The knee has been stored in a formaldehyde solution to conserve it. All experiments were performed in accordance with relevant guidelines and regulations.

## Data Availability

The datasets generated during and/or analyzed during the current study are available from the corresponding author on reasonable request.
